# Rural-Urban Differences in Informal Caregiving, Caregiving Intensity, and Health-Related Quality of Life

**DOI:** 10.1093/geroni/igaa057.814

**Published:** 2020-12-16

**Authors:** Steven Cohen, Julia McIlmail, Mary Greaney

**Affiliations:** University of Rhode Island, Kingston, Rhode Island, United States

## Abstract

Introduction Rural areas in the US have a disproportionately high population of older adults and have reduced access to services. Older adults in rural areas are more reliant on family and friends for care. However, little is known about rural-urban disparities among the 40+ million caregivers nationwide. As rural-urban health disparities are pervasive among older adults, there is a need to understand how rural-urban disparities impact caregiving experiences and health-related quality of life (HRQoL). The objectives of this study were to examine rural-urban differences caregiving, caregiving intensity (caregiving hours/week and types of care provided), and caregiver HRQoL. Methods Data were abstracted from the 2009 Behavioral Risk Factor Surveillance System (latest dataset to include county of residence and caregiver module). The primary measure of rural-urban status was Index of Relative Rurality (IRR) decile. Associations between rural-urban status and caregiving and rural-urban differences in caregiving intensity and HRQoL were examined using generalized linear models, controlling for confounding and accounting for complex sampling. Results Rural respondents were more likely to be caregivers than urban respondents (IRR decile OR=1.015, 95%CI 1.014-1.016). Rural caregivers, on average, provided 2.43 hours/week more caregiving for each one-decile increase in IRR decile (95%CI 2.34-2.52) and had worse overall HRQoL (OR=1.34, 95%CI 1.33-1.35). Conclusion Rural informal caregivers offer higher levels of care than urban counterparts, and increased caregiving in rural areas is associated with reduced HRQoL. These results can inform policies designed to improve caregiver health, and facilitate the translation of existing programs and interventions to address rural caregivers’ needs.

